# Mediational Effect of Fairburn’s Transdiagnostic Mechanisms Between Attachment to the Mother and Eating Disorder Symptoms in a Clinical Sample

**DOI:** 10.3389/fpsyg.2022.852977

**Published:** 2022-04-18

**Authors:** Laura Cortés-García, Carmen Martínez Calvo, Carmen Senra

**Affiliations:** ^1^PROMENTA Research Center, Department of Psychology, University of Oslo, Oslo, Norway; ^2^Unit of Eating Disorders, Provincial Hospital of Conxo, Santiago de Compostela, Spain; ^3^Department of Clinical Psychology and Psychobiology, Faculty of Psychology, University of Santiago de Compostela, Santiago de Compostela, Spain

**Keywords:** eating disorder, insecure attachment, transdiagnostic mechanisms, multiple mediation analysis, female patient

## Abstract

**Objective:**

Research has supported a link between insecure attachment and eating disorders (EDs); however, little is known about how this influence is exerted in young female EDs patients. This study tested, for the first time, a multiple mediational model, wherein the four Fairburn’s transdiagnostic mechanisms mediated the relationship between attachment to the mother and ED symptoms.

**Methods:**

A total of 101 female young EDs patients aged 15–24 were administered the Inventory of Parent and Peer Attachment, Eating Disorder Inventory-3 and Eating Attitudes Test-26 to assess attachment, the four transdiagnostic maintaining mechanisms and ED symptoms, respectively.

**Results:**

Comparison analyses showed that there were no significant differences between the diagnostic groups in terms of attachment and the transdiagnostic variables. Multiple mediational analyses indicated that low self-esteem and clinical perfectionism were significant mediators between insecure attachment to the mother and ED symptoms, while controlling for depressive symptoms.

**Conclusion:**

These findings suggest that the distal risk influence of insecure attachment to the mother in the development of ED symptoms might be explained by low self-esteem and high clinical perfectionism, controlling for depressive symptoms. Further investigation into the efficacy of cognitive-behavioral treatments targeting insecure attachment representations for young EDs patients is recommended.

## Introduction

Eating disorders (EDs) are highly distinctive psychiatric disorders characterized by severe and persistent disturbance in eating behaviors (American Psychiatric Association, 2013). EDs are associated with a wide variety of psychiatric and physical problems, and present high rates of persistence and recurrence, predominantly among adolescent and young adult women of Western societies (Ágh et al., 2016; Schmidt et al., 2016). Given the detrimental clinical and social impact of EDs, it is crucial to identify the factors that may contribute to their development and maintenance.

Insecure attachment has been recognized as a risk factor for the development of psychopathology, particularly EDs (Skarderud and Fonagy, 2012; Tasca, 2018). Individuals with insecure attachment are characterized by having experienced insensitive or unreliable care which favors the basis of representations of the self as unworthy or unlovable and of others as untrustworthy or unloving (Bowlby, 1969). However, even though insecure attachment, especially to mother, is associated with ED symptoms, this relationship is usually conditioned by the concurrence of other variables that channel the risk of early attachment experiences (Tasca and Balfour, 2014; Faber et al., 2018). Consequently, contemporary researchers have tried to identify intermediate mechanisms through which insecure attachment might influence the development of EDs symptoms (Cortés-García et al., 2019).

The transdiagnostic theory of the maintenance of EDs of Fairburn et al. (2003) postulates that there are four maintaining mechanisms interacting with the psychopathological core of EDs (i.e., overvaluation of the importance of weight and its control) and obstructing progress during treatment. These mechanisms are: (1) clinical perfectionism (i.e., a dysfunctional self-assessment system where personal worth is judged by effort and success in achieving very demanding goals related to diet and weight), (2) low self-esteem (i.e., negative self-image inherent to one’s own identity), (3) mood intolerance (i.e., inability to identify, express and cope with emotions), and (4) interpersonal difficulties.

Interestingly, insecure attachment has been consistently associated with such variables, that is, predicting the development of perfectionism (Wei et al., 2006; Ulu and Tezer, 2010), low self-esteem (Mikulincer and Shaver, 2012), inability to regulate emotions (Brenning and Braet, 2013), and interpersonal difficulties (Paech et al., 2016). Besides, due to the influence between these variables and EDs symptoms, their mediating role between insecure attachment and ED psychopathology has also been examined separately. In fact, previous studies showed that insecure attachment predicted the development and maintenance of unhealthy eating behaviors through clinical perfectionism (Dakanalis et al., 2014), low self-esteem (Shanmugam et al., 2012), emotion dysregulation (Jakovina et al., 2018; Pace et al., 2021), and interpersonal difficulties (Ty and Francis, 2013).

Thus far, it seems that both theory and empirical studies provide rationale to expect that insecure attachment, particularly to the mother, and the four Fairburn transdiagnostic mechanisms are associated with and partly explain ED symptoms (Fairburn et al., 2003; Tasca and Balfour, 2014). Furthermore, the role played by depressive symptoms in relation to insecure attachment (Dagan et al., 2018) and EDs (Puccio et al., 2016) cannot be neglected. On the one hand, the negative self-representations rooted in early insecure attachment relationships have a strong influence on the interpretation and response to future negative events, which in turn contributes to increased vulnerability to depression (Morley and Moran, 2011). On the other hand, depressive symptoms and disordered eating tend to co-occur (Puccio et al., 2016), and possibly, poor emotion regulation ingrained in individuals’ early mental representations (Malik et al., 2015; Faber et al., 2018) may increase the risk of both (Vögele et al., 2018). For instance, eating symptoms such as binge eating or overeating may serve as a strategy to modify or dampen negative emotions, such as depressive symptoms (Vögele et al., 2018). Thus, substantial evidence suggests that depressive symptoms may be both rooted on insecure attachment relationships (Morley and Moran, 2011) and participate in the etiology of EDs (Stice, 2001). In fact, it has been observed that depressive symptoms may mediate the association between insecure attachment and disordered eating (Cortés-García et al., 2019). In this regard, insecurely attached individuals, who presumably perceive themselves negatively, may be extra vulnerable to the multiple challenges through adolescence and emerging adulthood resulting in increased depressive symptoms and may find refuge in abnormal eating practices (Tasca, 2018). Thus, it can be assumed that when depressive symptoms are considered, the mediating effect of the maintaining mechanisms in the association between insecure attachment and EDs symptoms could be modified. To date, no study has investigated the links between insecure attachment to the mother, the Fairburn’s maintaining mechanisms and ED symptoms, while controlling for depressive symptoms, using clinical samples of young females. Accordingly, the aim of this study is two-fold: (1) to know how the quality of attachment to the mother and Fairburn’s maintenance mechanisms are related in a clinical sample of female with different ED diagnoses and (2) to examine whether insecure attachment to the mother contributes to the development of EDs symptoms through the maintenance mechanisms, while controlling for depressive symptoms. Given that this is the first study that investigates the mediational effect of Fairburn transdiagnostic mechanisms in a multiple mediation model among patients with EDs, our hypotheses are exploratory in nature based on existing knowledge. We speculate that there will be no differences between the different diagnoses with respect to maternal attachment and transdiagnostic mechanisms, and we hypothesize that the transdiagnostic mechanisms will mediate between maternal attachment and eating symptoms, even controlling for the effect of depressive symptoms.

## Materials and Methods

### Procedure and Participants

The study included 101 patients who consecutively accessed the Eating Disorders Unit of the Provincial Hospital of Conxo (Spain), and met the inclusion criteria (i.e., primary diagnosis of ED, being younger than 24 years old and female). The diagnoses were made following the Structured Clinical Interview for Axis 1 diagnoses of the DSM-IV-TR (First et al., 2002), which was administered by staff clinical psychologists. Concretely, 35 patients had a diagnosis of Anorexia Nervosa- restrictive subtype, 17 of Anorexia Nervosa-compulsive subtype, 31 of Bulimia Nervosa and 10 of ED-Non-Otherwise Specified according to the DSM-IV-TR (American Psychiatric Association, 2000). Eight patients were excluded from the investigation because they did not complete all questionnaires. Ages ranged from 15 to 24, with a mean of 17.81 (*SD* = 1.9) and 93% of participants were Caucasian, 5% Latino, and 2% Asian.

All procedures were approved by the Bioethics Committee at the University of Santiago de Compostela (Spain) before data collection commenced. Informed, written consent was obtained from the patients after being provided with a full description of the study. Before receiving any form of treatment, all patients completed the battery of self-report measures, under the supervision of a member of staff.

### Measures

The Inventory of Parent and Peer Attachment (Armsden and Greenberg, 1987) is a self-report measure of perceptions of the quality of attachment toward mother, father, and peers. For the present study, only the mother attachment scale was used; specifically, the revised version of 25 items, rated on a 5-point scale (from 1 = never to 5 = always). The overall score of attachment is obtained by summing responses of two subscales: degree of mutual trust and quality of communication, and by subtracting the score of the subscale of anger and alienation. Higher scores on trust and communication and lower score on alienation indicate higher attachment security. The Spanish-language version of the IPPA (Pardo et al., 2006) used in this study has shown satisfactory internal consistency and concurrent validity. In our sample, α coefficients ranged from 0.71 to 0.90: (a) trust (Cronbach’s α = 0.89), (b) communication (α = 0.90), and (c) alienation (α = 0.71).

The Eating Disorder Inventory-3 (Garner et al., 1983) is a self-report questionnaire used to assess the presence of EDs. Each item is scored on a 6-point scale and the score for each subscale is then summed. For this study, we only took the measures of the subscales’ low self-esteem (α = 0.59), perfectionism (α = 0.79), interpersonal distrust (α = 0.67), and interoceptive awareness (α = 0.75).

The Eating Attitudes Test-26 (EAT-26; Garner and Garfinkel, 1979) is a self-report questionnaire that assesses symptoms and concerns that are characteristic of EDs. It comprises 26 items that refer to thoughts, feelings, and behaviors common in EDs. Items are rated on a Likert scale ranging from 1 (never) to 6 (always). Higher scores indicate more disturbed eating pathology. In our study, the reliability of the EAT-26 was α = 0.87.

The Beck Depression Inventory-II (Beck et al., 1996) is a 21-item, self-report rating inventory that measures characteristic attitudes and symptoms of depression. The Spanish version of the BDI-II used in this study has shown satisfactory internal consistency, test–retest reliability, and concurrent and convergent validity (Sanz et al., 2003). In our study, the reliability of the BDI-II was α = 0.86.

### Analytic Strategy

All analyses were conducted in IBM SPSS Statistics 26. Preliminary descriptive information and Pearson correlations were obtained between the main study variables (including depressive symptoms) and the demographic variables that were significantly related to the outcome variables, which were included in the mediational analyses as covariates. Additionally, differences between diagnostic groups in demographic and clinical variables were investigated using Kruskal–Wallis tests. Significant results found with this analysis were followed up by Bonferroni-corrected Mann–Whitney *U* tests as *post hoc* comparisons.

Multiple mediation analyses were conducted using the PROCESS macro (Hayes, 2018), to explore whether transdiagnostic mechanisms (mediators) could explain the effect of attachment to the mother on ED symptoms. All four mediators were tested simultaneously in the analysis with the total patient. Pairwise contrasts of the specific indirect effects involved in each mediator were calculated. The indirect effect provided by 20000 bootstrap samples was examined. In addition, a 95% bias-corrected confidence interval (BootCI) was used. Depressive symptoms were included as a covariate in the mediation analyses due to the high correlation with insecure attachment and EDs symptoms (Puccio et al., 2016; Dagan et al., 2018).

## Results

Descriptive statistics for and Pearson’s correlations between the main study variables are presented in Table 1. These analyses revealed that insecure attachment to the mother was significantly related to more ED symptoms, more maladaptive perfectionism, less interoceptive awareness, less self-esteem, and more depressive symptoms. However, there was no significant association between attachment and interpersonal distrust.

**TABLE 1:** Descriptive information for and Pearson’s correlations between main study variables (*n* = 93).

	M (SD)	1	2	3	4	5	6	7
Attachment	50.39 (16.32)	–						
Self-esteem	24.22 (3.84)	0.310**	–					
Perfectionism	1.12 (0.82)	−0.360**	−0.099	–				
Interoceptive awareness	1.04 (0.61)	−0.343**	−0.198	0.330**	–			
Interpersonal distrust	0.93 (0.51)	0.201	−0.001	0.009	−0.129	–		
Eating symptoms	35.30 (10.84)	−0.349**	−0.413**	0.477**	0.457**	0.023*	–	
Depressive symptoms	25.98 (9.54)	−0.354**	−0.299**	0.332**	0.443**	−0.372**	0.445**	–

**p < 0.01; *p < 0.05. M, mean; SD, standard deviation.

The comparison between the different diagnostic groups is showed in Table 2. The analysis showed that there were significant differences between the groups only in terms of age (*H* = 16.7; *p* < 0.001; η^2^ = 0.202; *post hoc* BN > AN restrictive subtype), therefore, age was included as a covariate in the mediation analyses.

**TABLE 2:** Group differences in demographic characteristics and main study variables.

Variables	AN restrictive subtype (*n* = 35)	AN compulsive subtype (*n* = 17)	BN (*n* = 30)	ED-NOS (*n* = 11)	Kruskal–Wallis H	*p*	*Post hoc* (Adjusted *p* = 0.008)
Age	16.89 (1.05)	17.71 (1.36)	18.97 (2.5)	17.73 (1.62)	16.719	0.001	BN > AN restrictive subtype
Attachment	53.16 (16.69)	49.06 (12.91)	47.38 (18.79)	51.53 (12.85)	1.778	0.620	
Self-esteem	23.40 (4.67)	25.47 (2.15)	24.73 (3.56)	23.45 (3.30)	6.756	0.080	
Perfectionism	1.24 (0.89)	1.22 (0.65)	1.06 (0.79)	0.74 (0.80)	4.079	0.253	
Interoceptive awareness	0.89 (0.44)	1.01 (0.51)	1.29 (0.77)	0.87 (0.58)	4.709	0.194	
Interpersonal distrust	0.95 (0.51)	0.94 (0.54)	0.86 (0.51)	1.07 (0.53)	1.398	0.706	

Multiple mediational analyses results are presented in Table 3. The model accounted for 21.9% of the variance for ED symptoms (*R*^2^ = 0.219). While controlling age, the total indirect effect of insecure attachment on eating symptoms through all the proposed mediators was significant. Specifically, the mediators reduced the non-standardized regression coefficient of insecure attachment on eating symptoms from −0.26 (*p* < 0.001) to −0.06 (*p* = 0.34), which reflected 77% [(−0.26 to −0.06)/ −0.26] of the association between attachment and eating symptoms. When examining the specific indirect effect of each mediator, all maintenance variables except interpersonal distrust presented significant values. When controlling for age along with for depressive symptoms, the model accounted for 29.8% of the variance for ED symptoms (*R*^2^ = 0.298). The total indirect effect of insecure attachment on eating symptoms through all the proposed mediators was significant. Specifically, the mediators reduced the non-standardized regression coefficient of insecure attachment on eating symptoms from −0.19 (*p* = 0.01) to −0.05 (*p* = 0.40), which reflected 74% [(−0.19 to −0.05)/ −0.19] of the association between attachment and eating symptoms. When examining the specific indirect effect of each mediator, only low self-esteem and clinical perfectionism presented significant values. The pairwise contrasts revealed that in both models the specific indirect effects of insecure attachment on eating symptoms *via* self-esteem and perfectionism were significantly different and greater to the indirect effect through interpersonal distrust.

**TABLE 3:** Results of multiple mediation analyses of the effect of attachment on eating disorder symptoms.

Mediation pathway	Point estimate	SE	95% CI		
			Lower	Upper	*R*^2^ change
**Insecure attachment → Transdiagnostic mechanisms → Eating symptoms**					
Total	−0.3044	0.0783	−0.4652	−0.1592	
Self-esteem	−0.0949	0.0390	−0.1792	−0.0281	0.073***
Perfectionism	−0.1475	0.0567	−0.2701	−0.0502	0.110***
Interoceptive awareness	−0.0691	0.0371	−0.1508	−0.0056	0.023
Interpersonal distrust	0.0071	0.0206	−0.0343	0.0526	0.001
**Contrasts**					
Self-esteem vs. Perfectionism	0.0526	0.0637	−0.0651	0.1864	
Self-esteem vs. Interoceptive awareness	−0.0258	0.0577	−0.1397	0.0886	
Self-esteem vs. Interpersonal distrust	−0.1019	0.0437	−0.1939	−0.0212	
Perfectionism vs. Interoceptive awareness	−0.0783	0.0728	−0.2312	0.0600	
Perfectionism vs. Interpersonal distrust	−0.1545	0.0590	−0.2791	−0.0480	
Interoceptive awareness vs. Interpersonal distrust	−0.0762	0.0440	−0.1668	0.0062	
**Insecure attachment → Transdiagnostic mechanisms → Eating symptoms controlling depressive symptoms**					
Total	−0.1318	0.0559	−0.2547	−0.0359	
Self-esteem	−0.0448	0.0244	−0.1015	−0.0073	0.058**
Perfectionism	−0.0665	0.0360	−0.1516	−0.0114	0.084***
Interoceptive awareness	−0.0236	0.0183	−0.0646	0.0056	0.013
Interpersonal distrust	0.0031	0.0114	−0.0184	0.0303	0.006
**Contrasts**					
Self-esteem vs. Perfectionism	0.0217	0.0380	−0.0457	0.1056	
Self-esteem vs. Interoceptive awareness	−0.0211	0.0297	−0.0864	0.0322	
Self-esteem vs. Interpersonal distrust	−0.0479	0.0253	−0.1053	−0.0068	
Perfectionism vs. Interoceptive awareness	−0.0428	0.0409	−0.1381	0.0224	
Perfectionism vs. Interpersonal distrust	−0.0696	0.0349	−0.1516	−0.0150	
Interoceptive awareness vs. Interpersonal distrust	−0.0267	0.0207	−0.0708	0.0106	

Completely standardized indirect effects are reported. R^2^ change when adding each mediator, while controlling for the rest of variables. *p < 0.05, **p < 0.01, ***p < 0.001.

## Discussion

Previous research has shown that insecure attachment increases the risk for the development of EDs (Skarderud and Fonagy, 2012; Tasca, 2018); however, such effect is often exerted through other intermediate variables (Cortés-García et al., 2019). In this regard, the four transdiagnostic mechanisms for EDs proposed by Fairburn et al. (2003) may be potential candidates for mediation due to their robust associations with both insecure attachment and ED symptoms (Tasca, 2018). However, to date, no study has tested simultaneously such mechanisms in a multiple mediation model using a clinical sample of young ED patients. To fill this gap, we analyzed, for the first time, the role that Fairburn’s transdiagnostic mechanisms may play as links between insecure attachment to the mother and ED symptoms among young female ED patients. In addition, given the strong association of depressive symptoms with the model variables, we also explored such connections by controlling for their effect.

As expected, we found evidence for insecure attachment to the mother to be significantly related to ED symptoms (Tasca, 2018). In addition, all transdiagnostic mechanisms, except interpersonal distrust, were related to ED symptoms, which is partially in line with our hypothesis and previously mentioned research (Mikulincer and Shaver, 2012). Furthermore, results from comparison analyses revealed that there were no differences between the different ED diagnostic groups in the variables studied. Thus, our findings suggest that all four mechanisms could be involved in the persistence of eating psychopathology, regardless of diagnosis, as postulated by Fairburn et al. (2003).

The results from the mediation analyses further extends previous findings by demonstrating a multiple pathway between insecure attachment and ED symptoms among ED patients. In particular, low self-esteem, clinical perfectionism and low interoceptive awareness were found to mediate this association. However, when controlling for depressive symptoms, the mediating effect of interoceptive awareness disappeared and only low self-esteem and clinical perfectionism remained as significant mediators. These findings suggest that insecurely attached ED patients may develop dysfunctional cognitive representations of themselves based on early experiences with their first caregiver that come to undermine their self-esteem construction (Skarderud and Fonagy, 2012; Faber et al., 2018). Moreover, this negative self-image may be accompanied by a dysfunctional self-evaluation scheme that emphasizes the achievement of unrealistic goals in highly valued areas, such as success at controlling eating, shape and weight, in order to reinforce their self-worth (Fairburn et al., 2003; Faber et al., 2018). As a result, individuals who overestimate the importance of the aesthetics of thinness may maintain unhealthy eating behaviors to counter their feelings of worthlessness, especially after failing their unrealistic goals, that derived from their insecure attachment representations (Tasca, 2018).

As regards low interoceptive awareness, when controlling for depressive symptoms, the mediating effect was no longer significant. Such results pinpoint to the possibility that the effect of low interoceptive awareness in the link insecure attachment–EDs symptoms may be mostly due to depressive symptoms −which frequently occur concomitantly with EDs (Puccio et al., 2016). Thus, a possibility of these results is that unmet attachment needs could lead to depressive symptoms (Dagan et al., 2018) which interfere with the ability to identify emotions and body signals (e.g., hunger, satiety) (Lackner and Fresco, 2016) and contributes to the maintenance of disordered eating. In this regard, ED symptoms may be maintained among insecurely attached patients as ways to modulate their negative mood along with the inability to identify and understand body signs. However, cross-sectional data of this kind prevents conclusions being drawn about the causal direction of relationships.

Contrary to our expectations, interpersonal distrust did not significantly connect insecure attachment with eating symptoms, even though problems in interpersonal relations are common among ED patients reporting insecure attachment patterns (O’Shaughnessy and Dallos, 2009; Faber et al., 2018). It is possible that this null finding may be due, at least in part, to the use of the interpersonal distrust subscale of the EDI to measure interpersonal difficulties. Concretely, this subscale assesses disinterest in establishing close relationships and a general feeling of alienation in relationships (Garner et al., 1983), processes that, although certainly related to interpersonal difficulties, may not strictly target the interpersonal problems to which Fairburn’s transdiagnostic mechanism referred to [e.g., excessive social comparison with other persons considered as referents (Bamford and Halliwell, 2009; Ty and Francis, 2013) or excessive sensitivity to social rejection (De Paoli et al., 2017a,b)].

Taken together, our findings suggest that some insecurely attached patients who perceive themselves as unlovable and unworthy may be more vulnerable to developing poor self-image and dysfunctional self-assessment schemas focused on achieving unrealistic high goals about controlling weight, eating, and body shape. Moreover, unmet attachment needs may influence the development of more disordered eating when depressive symptoms interact with low interoceptive awareness, which are more proximal factors that contribute to or maintain disordered eating (Stice, 2002; Fairburn et al., 2003).

Such findings encourage the integration of dysfunctional representations of the self, others and the world that stem from an insecure attachment as distal risk factors of EDs into the transdiagnostic model so that, along with more proximal maintaining factors, can be targeted in theoretical models. Likewise, such results are valuable in designing preventative and intervention efforts for individuals at risk. To date, only one case study has demonstrated the efficacy of a treatment for EDs integrating both attachment and cognitive-behavioral factors in two cases of bulimia nervosa and binge eating disorders (Szalai, 2016). However, more randomized controlled studies targeting insecure attachment representations and other cognitive-behavioral maintaining factors of EDs are needed.

The present study is limited by its small clinical sample size and by the inclusion of only female patients, who were mostly adolescents and Caucasian and, therefore, our results must be viewed with caution and cannot be generalized to the whole of the ED population. In addition, the cross-sectional nature of the study design limits the ability to draw causal conclusions. Future studies should examine our model prospectively.

## Conclusion

The present findings suggest that the distal risk influence of insecure attachment to the mother in the development of ED symptoms might be exerted through low self-esteem and high clinical perfectionism, controlling for depressive symptoms. Therefore, our study highlights the theoretical and practical importance of understanding eating disorders as the result of the interaction of proximal and distal risk factors for which longitudinal studies are highly recommended.

## Data Availability Statement

The data that support the findings of this study are available from the corresponding author upon reasonable request. The data are not publicly available due to privacy and ethical restrictions.

## Ethics Statement

The studies involving human participants were reviewed and approved by the Bioethics Committee at the University of Santiago de Compostela (Spain). Written informed consent to participate in this study was provided by the participants’ legal guardian/next of kin.

## Author Contributions

LC-G, CM, and CS: conceptualization and writing – review and editing. LC-G and CM: methodology and data curation. LC-G: formal analysis and writing – original draft preparation. All authors contributed and approved the final version of the manuscript for submission.

## Conflict of Interest

The authors declare that the research was conducted in the absence of any commercial or financial relationships that could be construed as a potential conflict of interest.

## Publisher’s Note

All claims expressed in this article are solely those of the authors and do not necessarily represent those of their affiliated organizations, or those of the publisher, the editors and the reviewers. Any product that may be evaluated in this article, or claim that may be made by its manufacturer, is not guaranteed or endorsed by the publisher.
